# Complete continuum of maternal health care and Its determinants among women who gave birth in the past year in Mogadishu, Somalia

**DOI:** 10.3389/fgwh.2026.1801598

**Published:** 2026-07-16

**Authors:** Sharmake Gaiye Bashir, Hibo Hassan Mohamed, Sumaya Abdi Ali, Muna Mohamed hassan, Abdiqani abdiqaadir Mohamed, Amina Adan Ahmed, Yakub Burhan Abdullahi, Yusuf Hared Abdi, Hiba Abdi Salad, Mohamed Sharif Abdi, Naima Ibrahim Ahmed, Nuradin Abdullahi Sheikh Rashid, Ahmed Abdinasir Abdulle, Gallad Dahir Hassan

**Affiliations:** 1 Faculty of Health Sciences and Tropical Medicine, Somali National University, Mogadishu, Somalia; 2 Center for Health Research and Innovation, Somali National University, Mogadishu, Somalia; 3 Faculty of Health Science, Salaam University, Mogadishu, Somalia; 4 Department of Public Health, Faculty of Graduate Studies and Research Somali National University, Mogadishu, Somalia

**Keywords:** antenatal care, health service utilization, maternal health continuum, skilled birth attendance, Somalia

## Abstract

**Background:**

Maternal mortality remains a critical global health challenge, particularly in fragile settings. The continuum of maternal healthcare—comprising antenatal care (ANC), skilled birth attendance (SBA), and postnatal care (PNC)—is essential for reducing preventable deaths. In Somalia, with a maternal mortality ratio of approximately 563 per 100,000 live births, evidence on completion of the continuum of care remains limited.

**Objective:**

To assess the completion of the maternal healthcare continuum and its determinants among women who gave birth in the past year in Mogadishu, Somalia.

**Methods:**

A community-based cross-sectional study was conducted among 422 women of reproductive age in Mogadishu including Internally displaced people (IDPs). Data were collected using structured interviewer-administered questionnaires. Completion of the continuum of care was defined as receipt of at least four antenatal care visits (ANC ≥ 4), facility delivery, and postnatal care within 48 h after delivery. Bivariate and multivariable logistic regression analyses were performed to identify factors associated with completion.

**Results:**

Overall, **18.2%** of women completed the maternal health continuum. In the multivariable analysis, maternal education remained a strong predictor, with significantly lower odds among women with primary (AOR = 0.10; 95% CI: 0.02–0.43), secondary (AOR = 0.21; 95% CI: 0.05–0.86), and no formal education (AOR = 0.12; 95% CI: 0.02–0.56) compared to those with higher education. Husband's education was also significant, as women whose partners had no formal education (AOR = 0.14; 95% CI: 0.03–0.58) or primary education (AOR = 0.22; 95% CI: 0.05–0.95) had reduced odds of completion. Higher household income (≥300 USD) (AOR = 3.61; 95% CI: 1.28–10.14) and gravidity (4–7 pregnancies) (AOR = 5.08; 95% CI: 1.17–22.09) were positively associated with completion.

**Conclusion:**

Although only **18.2%** of women in Mogadishu completed the maternal healthcare continuum, substantial inequities persist. Socioeconomic status, maternal and partner education, and reproductive history significantly influence care completion. Strengthening maternal health programs through targeted education, economic support, and integrated service delivery is essential to improve equitable access and continuity of care in fragile settings.

## Introduction

Maternal mortality and morbidity persist as critical global public health challenges, claiming the daily lives of approximately 830 women, with 99% of these deaths occurring in developing countries (1). The continuum of maternal healthcare, encompassing antenatal care (ANC), skilled birth attendance (SBA), and postnatal care (PNC), represents a cornerstone strategy for reducing preventable maternal and neonatal deaths (2). Evidence demonstrates that completion of the full continuum yields substantial mortality reductions. Systematic reviews have documented an 83% reduction in combined neonatal, perinatal, and maternal mortality risks when women receive integrated ANC, SBA, and PNC services. Effective linkage across this continuum is necessary to improve maternal, newborn, and child health outcomes in low- and middle-income countries (LMICs), with particularly significant reductions observed in neonatal and perinatal mortality (3).

Despite global recommendations emphasizing comprehensive care packages, focusing on completion of the entire continuum rather than isolated service contacts remains critical for health system effectiveness (4). Service dropout between sequential stages is a fundamental weakness in maternal health systems, particularly in resource-constrained settings (5). In sub-Saharan Africa, only 30% of women complete the recommended care package of ANC contacts, skilled delivery, and PNC, with completion rates ranging from 6.5% in Chad to 69.5% in Sierra Leone (6, 7). The dropout rates intensified progressively: 17.5% at ANC, escalating to 20.2% at SBA, and reaching 30.9% at PNC (8). In East African Community countries, dropouts from the maternal, newborn, and child healthcare continuum remain alarmingly high (3, 9).

Fragile and conflict-affected settings comprise these challenges through multiple intersecting vulnerabilities (10, 11). In sub-Saharan Africa, protracted insecurity, weak health infrastructure, large-scale displacement, endemic poverty, and pervasive gender inequalities systematically disrupt the continuity of care (7, 12, 13). These contextual factors create barriers spanning financial constraints, geographical distance, restrictive policies, and stigma, particularly affecting adolescent girls and marginalized populations (12).

Somalia exemplifies these complexities, with one of the world's highest maternal mortality ratios of 563 deaths per 100,000 live births, reflecting three decades of political instability, civil conflict, and protracted humanitarian crises (14–16). Within this context, Mogadishu, as Somalia's urban capital and home to the Banadir region, presents a paradoxical situation: relative service availability coexists with persistent health inequities, displacement pressures, and systemic fragility (15). Only 9.7% of the Somali women completed the recommended ANC4 + visits, 2.8% utilized skilled birth attendants, and 0.6% received all three maternal healthcare services (14).

Despite growing recognition of these challenges, community-based evidence on the completion of the full maternal healthcare continuum and its determinants remains critically limited in Somalia, particularly among women with recent births (14). Identifying these determinants is essential for evidence-informed policy development, targeted programmatic interventions, and accelerating progress toward Sustainable Development Goal 3 (11). Therefore, this study aimed to assess the complete continuum of maternal healthcare and its determinants among women who gave birth in the past year in Mogadishu, Somalia.

## Methods

### Study design and setting

This community-based cross-sectional study was conducted in Mogadishu, Somalia. This study targeted women of reproductive age who had experienced at least one live birth prior to the survey. Mogadishu is characterized by substantial urban–IDP heterogeneity, variable access to maternal health services, and a fragile health system, making it an appropriate setting for examining completion of the maternal health continuum of care.

### Study population and sampling

The study population comprised women of reproductive age who had delivered at least once and resided in the study area during the survey period.

A community-based sampling approach was employed to recruit eligible participants. The study area was stratified by type of residence [urban, rural, and internally displaced persons (IDP) camps] to capture population heterogeneity. Within each stratum, households were selected using a systematic sampling technique. In each selected household, one eligible woman who had experienced at least one live birth was identified and invited to participate. Where more than one eligible respondent was present, one was selected using simple random sampling. Data collection continued until the required sample size was achieved.

### Sample size determination

The sample size was calculated using the single population proportion formula, assuming a 95% confidence level, a 5% margin of error, and a conservative estimated proportion of 50% for completion of the maternal continuum of care. This assumption was used to ensure maximum sample size and adequate statistical power, given variations in definitions and limited comparability across existing studies.

$$n=\frac{Z^{2}\times p\times (1-p)}{d^{2}}$$

Where:

*Z* = 1.96 (standard normal value at 95% confidence level),
*p* = 0.50 (assumed proportion),
*d* = 0.05 (margin of error).
  $$n=\frac{(1.96{)}^{2}\;\times 0.5\;\times 0.5}{{\left(0.05\right)}^{2}}=384$$

To account for potential non-response and incomplete data, a 10% contingency was added, resulting in a final sample size of **422 participants**.

However, 15 observations were subsequently dropped from the regression analyses due to multicollinearity and perfect prediction among certain variables. Therefore, a total of 407 observations were retained and included in the bivariate and multivariable logistic regression analyses.

### Data collection and variables

Data were collected using a structured interviewer-administered questionnaire by trained data collectors. Information on sociodemographic characteristics, reproductive history, socioeconomic status, and utilization of maternal health services was obtained.

Key maternal health service variables were operationalized as follows: antenatal care (ANC ≥ 4) was defined as attending four or more ANC visits during the most recent pregnancy; facility delivery was defined as childbirth occurring in a recognized health facility with skilled attendance; and postnatal care (PNC) was defined as receiving care within 48 h after delivery, in line with WHO recommendations. Women who did not attend any antenatal care were classified as not meeting the ANC ≥ 4 criterion. Thus, the ANC variable was coded to include all participants, However, some observations had incomplete information on antenatal care visits. Women with missing data required to construct the continuum of care (CoC) variable were excluded from the analysis.

### Outcome variable: completion of continuum of maternal health care

The primary outcome was completion of the continuum of maternal health care, defined as a binary variable. Women were classified as having completed the continuum of care (Yes) if they reported the following:

1. Attendance of **four or more antenatal care (ANC** **≥** **4) visits** during the most recent pregnancy,
2. Delivery at a health facility (skilled birth attendance), and
3. Receipt of **postnatal care (PNC) within 48 h after delivery**.

Women who did not meet all three criteria were categorized as **No (not completed)**.

### Independent variables

The independent variables included maternal age, marital status, mother's education level, husband's education level, mother's occupation, average monthly household income, place of residence, gravidity, parity, and whether the last pregnancy was planned.

For analysis, selected variables were **categorized** as follows: maternal age (15–19, 20–24, 25–29, 30–34, 35–39, ≥40 years), gravidity (1–3, 4–7, ≥8), parity (1–3, 4–7, ≥8 live births), and household income (<100 USD, 100–199 USD, 200–299 USD, ≥300 USD, and do not know). All variables were coded prior to analysis.

### Conceptual framework

This study was guided by a conceptual framework adapted from the Andersen Behavioral Model of Health Service Utilization, which categorizes determinants of healthcare use into predisposing, enabling, and need factors. Sociodemographic characteristics such as maternal age, education, and marital status were considered predisposing factors; household income, place of residence, and partner's education were treated as enabling factors; while reproductive characteristics such as parity and gravidity were included as need-related factors. This framework informed the selection of independent variables and the analysis of factors associated with completion of the maternal continuum of care.

### Handling of missing data

Observations with incomplete information required to construct the continuum of care (CoC) variable, including antenatal care visits, facility delivery, and postnatal care within 48 h, were excluded from the analysis. Although facility delivery and postnatal care variables were complete, some women lacked information on the number of antenatal care visits. Consequently, these observations could not be classified for CoC completion. In addition, records with missing values in key independent variables were removed using a complete-case approach. As a result, a total of 407 complete cases were included in the final bivariate and multivariable analyses.

### Statistical analysis

Data were analyzed using Stata. Descriptive statistics were used to summarize participant characteristics, and categorical variables are presented as frequencies and percentages.

The outcome variable, completion of the continuum of care (CoC), was constructed as a composite binary variable, defined by the combined achievement of ANC ≥ 4 visits, facility delivery, and postnatal care within 48 h.

Bivariate analysis was performed using Pearson's chi-square test and logistic regression to estimate crude odds ratios (CORs) with 95% confidence intervals (CIs). All independent variables were included in the multivariable logistic regression model regardless of their statistical significance at the bivariate level, based on their epidemiological relevance and theoretical importance. Adjusted odds ratios (AORs) with 95% confidence intervals (CIs) were reported. Statistical significance was set at *p* < 0.05.

### Ethical considerations

All participants provided informed consent before participation. Data were collected anonymously and confidentiality was strictly maintained throughout data collection, analysis, and reporting. The study was conducted in accordance with the Declaration of Helsinki and the applicable local ethical guidelines.

## Result

Table 1 summarizes the sociodemographic and obstetric characteristics of the 422 mothers included in the study. Most respondents were aged 20–29 years (54.8%) and married (80.3%). A large proportion of mothers had no formal education (61.4%) and most were housewives (71.1%). Nearly half of the households (45.5%) reported a monthly income below USD 100. More than half of the mothers resided in urban areas (51.9%), while 41.5% lived in IDP camps. Regarding reproductive history, 46.7% of respondents had experienced one to three pregnancies, and 48.6% had one to three live births. Most mothers reported that their most recent pregnancy was unplanned (61.4%).

**Table 1:** Socio-demographic and obstetric characteristics of mothers who gave birth in the last year in IDP camps in Mogadishu, Somalia (*N* = 422).

Characteristic	Category	Frequency (%)
Age of mother (years)	15–19	40 (9.5)
	20–24	113 (26.8)
	25–29	118 (28.0)
	30–34	84 (19.9)
	35–39	53 (12.6)
	≥40	14 (3.3)
Marital status	Married	339 (80.3)
	Divorced/Separated	54 (12.8)
	Widowed	29 (6.9)
Mother's education level	No formal education	259 (61.4)
	Primary	73 (17.3)
	Secondary	51 (12.1)
	College/University	39 (9.2)
Husband's education level	No formal education	193 (45.7)
	Primary	65 (15.4)
	Secondary	56 (13.3)
	College/University	86 (20.4)
	Not applicable	22 (5.2)
Mother's occupation	Housewife	300 (71.1)
	Self-employed	65 (15.4)
	Employed	52 (12.3)
	Student	5 (1.2)
Average monthly household income (USD)	<100	192 (45.5)
	100–199	88 (20.9)
	200–299	66 (15.6)
	≥300	55 (13.0)
	Do not know	21 (5.0)
Place of residence	Urban	219 (51.9)
	IDP camp	175 (41.5)
	Rural	28 (6.6)
Gravidity	1–3 pregnancies	197 (46.7)
	4–7 pregnancies	146 (34.6)
	≥8 pregnancies	79 (18.7)
Parity (live births)	1–3 live births	205 (48.6)
	4–7 live births	171 (40.5)
	≥8 live births	46 (10.9)
Last pregnancy planned	Yes	163 (38.6)
	No	259 (61.4)

**Figure 1:**
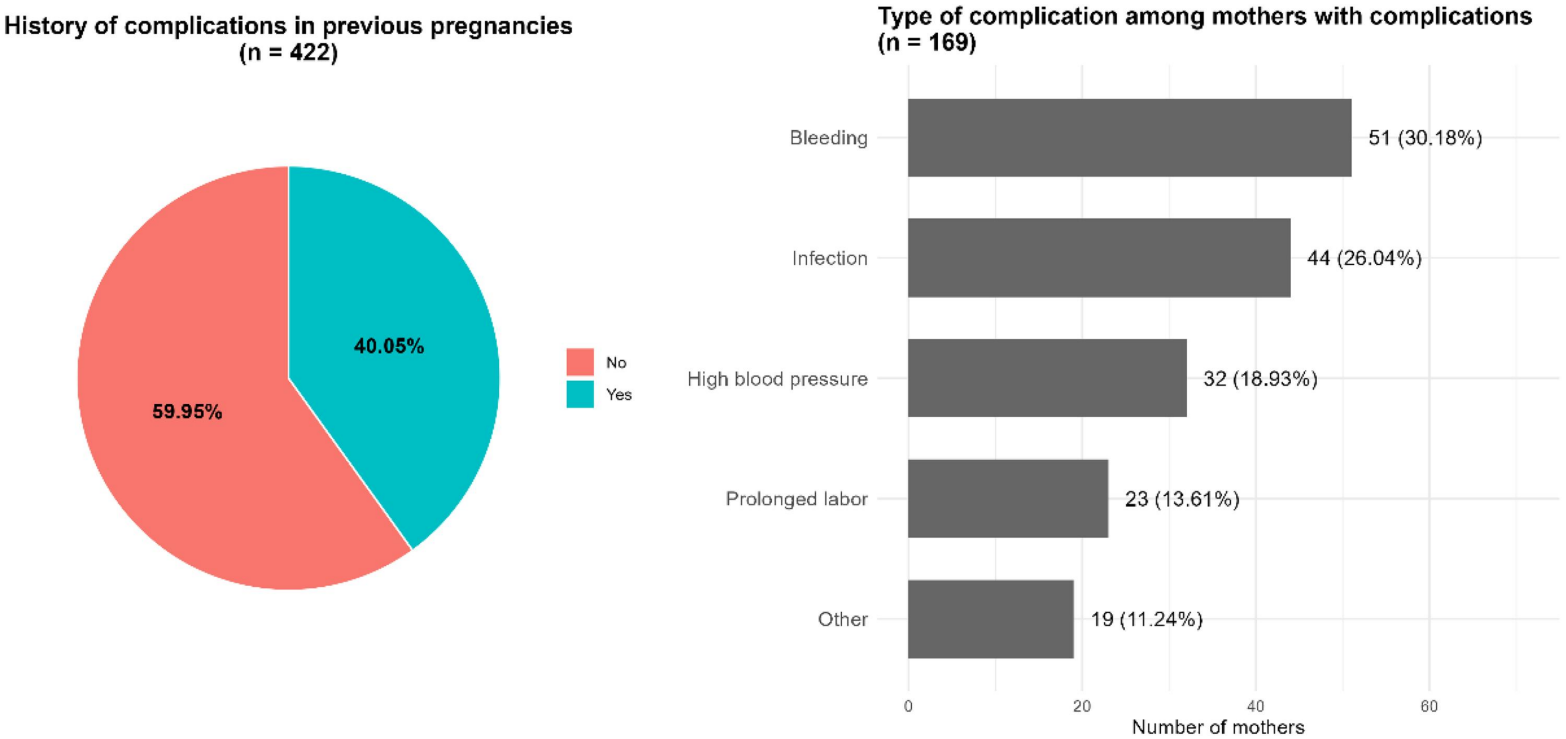
History and types of complications in previous pregnancies among mothers who gave birth in the last year in IDP camps in Mogadishu, Somalia.

Figure 1 shows that 40.1% of the mothers reported a history of complications in previous pregnancies, while 59.9% reported no such history. Among the mothers who experienced complications (*n* = 169), bleeding was the most commonly reported complication (30.2%), followed by infection (26.0%), and high blood pressure (18.9%). Prolonged labor accounted for 13.6% of the reported complications, while other complications accounted for 11.2%.

Table 2 presents antenatal care (ANC) utilization and related characteristics among the 422 mothers included in the study. Overall, 70.9% of mothers reported attending ANC during their most recent pregnancy. Among those who did not attend ANC, the most frequently reported reason was lack of money (39.0%), followed by perceiving ANC as unnecessary (29.3%) and long distance to health facilities (22.0%). Among ANC attendees, half initiated care during the first trimester (50.5%), while 33.1% and 16.4% initiated ANC in the second and third trimesters, respectively. Regarding the number of visits, 42.1% attended two to three ANC visits, and 28.1% attended four or more visits. Additionally, 63.6% of ANC attendees reported being informed about pregnancy danger signs during their ANC visits.

**Table 2:** Antenatal care utilization and characteristics among mothers who gave birth in the last year in Mogadishu, Somalia.

Indicator	Category	*n* (%)
Attended ANC during last pregnancy (*N* = 422)	Yes	299 (70.85)
	No	123 (29.15)
Main reason for not attending ANC (among non-attendees, *n* = 123)	Lack of money	48 (39.02)
	Did not feel it was necessary	36 (29.27)
	Facility too far	27 (21.95)
	No permission/support	4 (3.25)
	Other	8 (6.50)
Timing of ANC initiation (among ANC attendees, *n* = 299)	First trimester	151 (50.50)
	Second trimester	99 (33.11)
	Third trimester	49 (16.39)
Number of ANC visits attended (among ANC attendees, *n* = 299)	1 visit	89 (29.77)
	2–3 visits	126 (42.14)
	≥4 visits	84 (28.09)
Informed about pregnancy danger signs during ANC (among ANC attendees, *n* = 299)	Yes	190 (63.55)
	No	109 (36.45)

Table 3 summarizes the delivery care practices and complications among mothers who gave birth in the previous year. Nearly two-thirds of deliveries occurred in health facilities (62.6%), while 37.4% occurred at home. Skilled health workers assisted the majority of deliveries (63.0%), 26.3% were assisted by traditional birth attendants, and 10.7% by relatives or friends. Among mothers who delivered at home, the most commonly reported reasons were cost (25.3%) and cultural preference (24.7%), followed by distance to health facilities (19.6%) and lack of transport (10.1%). Overall, 49.1% of the mothers reported experiencing complications during delivery.

**Table 3:** Delivery care practices and complications among mothers who gave birth in the last year in Mogadishu, Somalia.

Indicator	Category	*n* (%)
Place of delivery (*N* = 422)	Health facility	264 (62.56)
	Home	158 (37.44)
Type of delivery assistance (*N* = 422)	Skilled health worker (doctor/nurse/midwife)	266 (63.03)
	Traditional birth attendant	111 (26.30)
	Relative/friend	45 (10.66)
Main reason for home delivery (among home deliveries, *n* = 158)	Cost	40 (25.32)
	Cultural preference	39 (24.68)
	Facility too far	31 (19.62)
	No transport	16 (10.13)
	Sudden labor	8 (5.06)
	Other	24 (15.19)
Experienced complications during delivery (*N* = 422)	Yes	207 (49.05)
	No	215 (50.95)

Table 4 presents postnatal care within 48 h after delivery (PNC) utilization and timing among the study participants. Overall, 65.9% of mothers reported receiving postnatal care after delivery, while 34.1% did not. Among those who did not receive PNC, the most commonly reported reason was lack of awareness of postnatal care services (37.5%), followed by distance to health facilities (24.3%) and cost (21.5%).

**Table 4:** Postnatal care utilization and timing among mothers who gave birth in the last year in Mogadishu, Somalia.

Indicator	Category	*n* (%)
Received postnatal care within 48 h after delivery (*N* = 422)	Yes	278 (65.88)
	No	144 (34.12)
Main reason for not receiving PNC (among non-recipients, *n* = 144)	Not aware of PNC	54 (37.50)
	Facility too far	35 (24.31)
	Cost	31 (21.53)
	Felt healthy	10 (6.94)
	Other	14 (9.72)

Table 5 summarizes the newborn care practices of the mothers included in this study. Approximately two-thirds of the newborns were checked by a health professional within 24 h of birth (66.1%). Early initiation of breastfeeding within one hour after birth was reported by 76.8% of the mothers. Immediate care after birth was provided for 68.0% of newborns, while 32.0% did not receive such care. Among those who did not receive immediate care, home delivery was the most frequently reported reason (60.0%), followed by a lack of awareness (21.5%) and cultural beliefs (5.2%).

**Table 5:** Newborn care practices among mothers who gave birth in the last year in Mogadishu, Somalia.

Indicator	Category	*n* (%)
Newborn checked by a health professional within 24 h (*N* = 422)	Yes	279 (66.11)
	No	143 (33.89)
Breastfeeding initiated within one hour after birth (*N* = 422)	Yes	324 (76.78)
	No	98 (23.22)
Newborn received immediate care after birth (*N* = 422)	Yes	287 (68.01)
	No	135 (31.99)
Reason newborn did not receive immediate care (*n* = 135)	Home delivery	81 (60.00)
	Lack of awareness	29 (21.48)
	Cultural beliefs	7 (5.19)
	Other	18 (13.33)

Table 6 presents barriers and perceptions related to the completion of maternal and newborn health services. Among mothers who reported barriers to completing services (*n* = 124), the most commonly cited barrier was cost of services (31.5%), followed by lack of awareness (28.2%) and cultural or religious beliefs (16.1%). Distance or transport challenges (9.7%), husband or family decision-making (8.9%), and perceived poor quality of care (5.7%) were less frequently reported. Overall, 76.5% of mothers believed that completing all maternal health services is important, and 71.8% reported that they would recommend maternal health services to other women.

**Table 6:** Barriers and perceptions related to completion of maternal and newborn health services among mothers in Mogadishu, Somalia.

Indicator	Category	*n* (%)
Main barriers to completing maternal health services (*n* = 124)	Cost of services	39 (31.45)
	Lack of awareness	35 (28.23)
	Cultural/religious beliefs	20 (16.13)
	Distance/transport	12 (9.68)
	Husband/family decision	11 (8.87)
	Poor quality of care	7 (5.65)
Belief that completing all maternal health services is important (*N* = 422)	Yes	323 (76.54)
	No	99 (23.46)
Would recommend maternal health services to other women (*N* = 422)	Yes	303 (71.80)
	No	119 (28.20)

**Figure 2:**
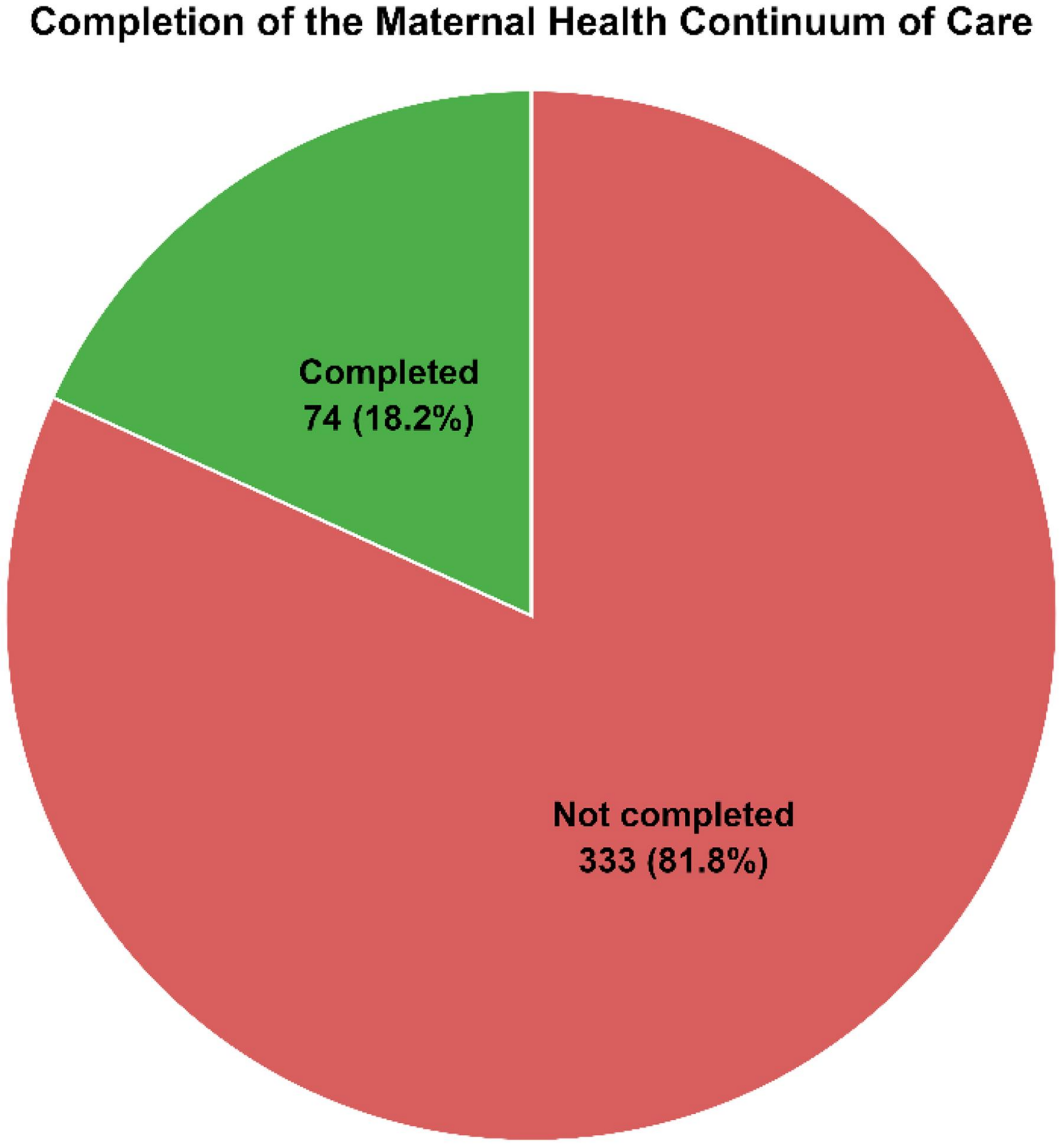
Completion of the maternal health continuum of care among mothers who gave birth in the last year in Mogadishu, Somalia.

Figure 2 illustrates the distribution of completion of the maternal health continuum of care among women in Mogadishu. Overall, a substantial majority of women (81.8%) did not complete the continuum of care, while only 18.2% achieved full completion.

Table 7 presents the bivariate associations between selected socio-demographic, economic, and reproductive factors and completion of the maternal health continuum of care. Maternal age was not significantly associated with completion of care overall; however, mothers aged ≥40 years had higher odds of completion compared with those aged 15–19 years (COR = 5.93; 95% CI: 1.18–29.68). Marital status was not significantly associated with completion, although widowed and divorced/separated women showed lower odds compared to married women. Both maternal and husband's education levels were strongly associated with continuum completion. Mothers and husbands with lower levels of education had significantly reduced odds of completing the continuum compared to those with college or university education (*p* < 0.001). Household income showed a significant association with completion. Women from higher-income households (≥300 USD) had significantly higher odds of completing the continuum (COR = 5.96; 95% CI: 2.68–13.25), while those in lower income categories (<200 USD) had substantially reduced odds. Place of residence was also significantly associated with completion. Urban residents had markedly higher odds of completing the continuum compared with women living in IDP camps (COR = 9.08; 95% CI: 4.21–19.56), while rural residence was not significantly different. Gravidity and parity were not significantly associated with completion of care. Although women with ≥8 births or pregnancies showed lower odds, these associations were not statistically significant. Maternal occupation showed limited association overall, although students had higher odds of completion compared to employed women (COR = 7.17; 95% CI: 1.04–49.28), based on a small sample. Finally, women whose last pregnancy was planned had significantly higher odds of completing the continuum of care compared to those with unplanned pregnancies (COR = 2.23; 95% CI: 1.34–3.72).

**Table 7:** Bivariate analysis of factors associated with completion of continuum of maternal health care among mothers in Mogadishu, Somalia (*N* = 407).

Variable	Category	CoC No *n* (%)	CoC Yes *n* (%)	Crude OR (95% CI)	*p*-value
Age of mother (years)	15–19 (Ref)	32 (91.43)	3 (8.57)	1.00	—
	20–24	85 (77.27)	25 (22.73)	3.14 (0.89–11.11)	0.076
	25–29	92 (80.00)	23 (20.00)	2.67 (0.75–9.48)	0.130
	30–34	70 (84.34)	13 (15.66)	1.98 (0.53–7.44)	0.311
	35–39	45 (90.00)	5 (10.00)	1.19 (0.26–5.32)	0.824
	≥40	9 (64.29)	5 (35.71)	5.93 (1.18–29.68)	0.030
Marital status	Married (Ref)	258 (79.38)	67 (20.62)	1.00	—
	Divorced/Separated	48 (90.57)	5 (9.43)	0.40 (0.15–1.05)	0.062
	Widowed	27 (93.10)	2 (6.90)	0.29 (0.07–1.23)	0.092
Mother's education level	College/University (Ref)	8 (21.05)	30 (78.95)	1.00	—
	Primary	61 (85.92)	10 (14.08)	0.04 (0.02–0.12)	<0.001
	Secondary	32 (64.00)	18 (36.00)	0.15 (0.06–0.40)	<0.001
	No formal education	232 (93.55)	16 (6.45)	0.02 (0.01–0.05)	<0.001
Husband's education level	College/University (Ref)	35 (41.67)	49 (58.33)	1.00	—
	No formal education	176 (95.14)	9 (4.86)	0.04 (0.02–0.08)	<0.001
	Primary	56 (91.80)	5 (8.20)	0.06 (0.02–0.18)	<0.001
	Secondary	47 (85.45)	8 (14.55)	0.12 (0.05–0.29)	<0.001
	Not applicable	19 (86.36)	3 (13.64)	0.11 (0.03–0.41)	0.001
Mother's occupation	Employed (Ref)	43 (82.69)	9 (17.31)	1.00	—
	Student	2 (40.00)	3 (60.00)	7.17 (1.04–49.28)	0.045
	Housewife	233 (81.75)	52 (18.25)	1.07 (0.49–2.32)	0.872
	Self-employed	55 (84.62)	10 (15.38)	0.87 (0.32–2.33)	0.779
Average monthly household income	200–299 USD (Ref)	44 (70.97)	18 (29.03)	1.00	—
	100–199 USD	80 (94.12)	5 (5.88)	0.15 (0.05–0.44)	<0.001
	<100 USD	174 (94.05)	11 (5.95)	0.15 (0.07–0.35)	<0.001
	Do not know	19 (95.00)	1 (5.00)	0.13 (0.02–1.03)	0.054
	≥300 USD	16 (29.09)	39 (70.91)	5.96 (2.68–13.25)	<0.001
Place of residence	IDP camp (Ref)	162 (95.29)	8 (4.71)	1.00	—
	Rural	26 (96.30)	1 (3.70)	0.78 (0.09–6.49)	0.817
	Urban	145 (69.05)	65 (30.95)	9.08 (4.21–19.56)	<0.001
Gravidity	1–3 (Ref)	156 (82.54)	33 (17.46)	1.00	—
	4–7	110 (78.01)	31 (21.99)	1.33 (0.77–2.30)	0.305
	≥8	67 (87.01)	10 (12.99)	0.71 (0.33–1.51)	0.370
Parity (live births)	1–3 live births (Ref)	159 (80.71)	38 (19.29)	1.00	—
	4–7 live births	134 (81.21)	31 (18.79)	0.97 (0.57–1.64)	0.904
	≥8 live births	40 (88.89)	5 (11.11)	0.52 (0.19–1.41)	0.202
Last pregnancy planned	No (Ref)	214 (86.64)	33 (13.36)	1.00	—
	Yes	119 (74.38)	41 (25.62)	2.23 (1.34–3.72)	0.002

Table 8 presents the results of the multivariable logistic regression analysis identifying factors independently associated with completion of the maternal health continuum of care. After adjustment for potential confounders, maternal education remained a significant predictor. Mothers with primary (AOR = 0.10; 95% CI: 0.02–0.43), secondary (AOR = 0.21; 95% CI: 0.05–0.86), and no formal education (AOR = 0.12; 95% CI: 0.02–0.56) had significantly lower odds of completing the continuum compared with those with college or university education. Husband's education was also independently associated. Women whose husbands had no formal education (AOR = 0.14; 95% CI: 0.03–0.58) or primary education (AOR = 0.22; 95% CI: 0.05–0.95) had significantly lower odds of completing the continuum compared to those whose husbands had higher education. Household income showed a significant association, with women from higher-income households (≥300 USD) having increased odds of completing the continuum of care (AOR = 3.61; 95% CI: 1.28–10.14). Gravidity was also significantly associated, as women with 4–7 pregnancies had higher odds of completing the continuum compared to those with 1–3 pregnancies (AOR = 5.08; 95% CI: 1.17–22.09). Maternal occupation showed a partial association, with self-employed women having lower odds of completing the continuum compared to employed women (AOR = 0.13; 95% CI: 0.02–0.80). In contrast, maternal age, marital status, place of residence, parity, and pregnancy intention were not significantly associated with completion of the continuum of care in the adjusted model.

**Table 8:** Multivariable logistic regression analysis of factors associated with completion of the maternal health continuum of care among women in Mogadishu, Somalia (*N* = 407).

Variable	Category	CoC No *n* (%)	CoC Yes *n* (%)	Adjusted OR (95% CI)	*p*-value
Age of mother (years)	15–19 (Ref)	32 (91.43)	3 (8.57)	1.00	—
	20–24	85 (77.27)	25 (22.73)	0.63 (0.11–3.53)	0.601
	25–29	92 (80.00)	23 (20.00)	0.61 (0.10–3.53)	0.576
	30–34	70 (84.34)	13 (15.66)	0.33 (0.05–2.41)	0.276
	35–39	45 (90.00)	5 (10.00)	0.44 (0.05–3.99)	0.469
	≥40	9 (64.29)	5 (35.71)	4.08 (0.41–40.65)	0.230
Marital status	Married (Ref)	258 (79.38)	67 (20.62)	1.00	—
	Divorced/Separated	48 (90.57)	5 (9.43)	1.43 (0.34–6.02)	0.628
	Widowed	27 (93.10)	2 (6.90)	0.46 (0.06–3.35)	0.443
Mother's education level	College/University (Ref)	8 (21.05)	30 (78.95)	1.00	—
	Primary	61 (85.92)	10 (14.08)	0.10 (0.02–0.43)	0.002
	Secondary	32 (64.00)	18 (36.00)	0.21 (0.05–0.86)	0.030
	No formal education	232 (93.55)	16 (6.45)	0.12 (0.02–0.56)	0.008
Husband's education level	College/University (Ref)	35 (41.67)	49 (58.33)	1.00	—
	No formal education	176 (95.14)	9 (4.86)	0.14 (0.03–0.58)	0.007
	Primary	56 (91.80)	5 (8.20)	0.22 (0.05–0.95)	0.042
	Secondary	47 (85.45)	8 (14.55)	0.34 (0.10–1.20)	0.094
	Not applicable	19 (86.36)	3 (13.64)	0.68 (0.09–4.96)	0.707
Mother's occupation	Employed (Ref)	43 (82.69)	9 (17.31)	1.00	—
	Student	2 (40.00)	3 (60.00)	0.36 (0.01–9.21)	0.538
	Housewife	233 (81.75)	52 (18.25)	0.75 (0.17–3.40)	0.709
	Self-employed	55 (84.62)	10 (15.38)	0.13 (0.02–0.80)	0.028
Average monthly household income	200–299 USD (Ref)	44 (70.97)	18 (29.03)	1.00	—
	100–199 USD	80 (94.12)	5 (5.88)	0.30 (0.08–1.11)	0.072
	<100 USD	174 (94.05)	11 (5.95)	0.28 (0.06–1.32)	0.108
	Do not know	19 (95.00)	1 (5.00)	0.11 (0.01–1.19)	0.069
	≥300 USD	16 (29.09)	39 (70.91)	3.61 (1.28–10.14)	0.015
Place of residence	IDP camp (Ref)	162 (95.29)	8 (4.71)	1.00	—
	Rural	26 (96.30)	1 (3.70)	0.54 (0.04–6.97)	0.637
	Urban	145 (69.05)	65 (30.95)	0.93 (0.19–4.46)	0.927
Gravidity	1–3 (Ref)	156 (82.54)	33 (17.46)	1.00	—
	4–7	110 (78.01)	31 (21.99)	5.08 (1.17–22.09)	0.030
	≥8	67 (87.01)	10 (12.99)	4.16 (0.62–28.17)	0.144
Parity (live births)	1–3 live births (Ref)	159 (80.71)	38 (19.29)	1.00	—
	4–7 live births	134 (81.21)	31 (18.79)	0.55 (0.13–2.34)	0.417
	≥8 live births	40 (88.89)	5 (11.11)	0.45 (0.06–3.69)	0.460
Last pregnancy planned	No (Ref)	214 (86.64)	33 (13.36)	1.00	—
	Yes	119 (74.38)	41 (25.62)	2.19 (0.96–4.99)	0.063

## Discussion

This study found that only 18.2% of women in Mogadishu who gave birth in the past year completed the full continuum of maternal health care, defined as receiving antenatal care (ANC ≥ 4), facility delivery, and postnatal care within 48 h. After adjusting for sociodemographic and reproductive factors, several determinants remained independently associated with continuum completion. Maternal education emerged as a strong predictor, with significantly lower odds of completion among women with primary (AOR = 0.10; 95% CI: 0.02–0.43), secondary (AOR = 0.21; 95% CI: 0.05–0.86), and no formal education (AOR = 0.12; 95% CI: 0.02–0.56) compared to those with higher education. Similarly, husband's education was significantly associated, as women whose partners had no formal education (AOR = 0.14; 95% CI: 0.03–0.58) or primary education (AOR = 0.22; 95% CI: 0.05–0.95) were less likely to complete the continuum. Higher household income (≥300 USD) was associated with increased odds of completion (AOR = 3.61; 95% CI: 1.28–10.14), while women with moderate gravidity (4–7 pregnancies) also demonstrated higher odds (AOR = 5.08; 95% CI: 1.17–22.09) compared to those with lower gravidity. These findings highlight the critical role of socioeconomic status, educational attainment, and reproductive experience in shaping maternal healthcare utilization in fragile urban settings.

The observed completion rate of 18.2% in this Mogadishu study is consistent with, though slightly below, the pooled regional average for sub-Saharan Africa, which has been estimated at 18.7% to 25% across multi-country analyses (4, 17). East African countries typically report completion rates between 17.5% and 17.9% (18), while more recent studies from Ethiopia and Nigeria have documented rates as low as 6.5%–12.5% (6). The **18.2%** completion rate in Mogadishu is lower than the levels observed in Southern African settings, where rates range from 38% to 81.4% (17, 19). This low completion rate likely reflects the impact of protracted conflict, displacement pressures, and health system fragility, despite the urban capital's relatively greater concentration of health facilities compared to rural or other conflict-affected regions of Somalia and East Africa. However, the findings also highlight marked within-city inequities, as 41.5% of the study population resided in internally displaced persons (IDP) camps, where service access remains constrained. The association between planned pregnancy and continuum completion aligns with broader evidence from sub-Saharan Africa, demonstrating that pregnancy intention significantly influences maternal healthcare utilization (20), a relationship that persists even after adjusting for socioeconomic and demographic confounders.

Pregnancy intention reflects maternal reproductive autonomy and readiness, which are factors that strongly influence health-seeking behaviors throughout the maternal care pathway (20). Women who plan their pregnancies are more likely to initiate antenatal care early, prepare logistically and financially for facility delivery, and prioritize postnatal follow-up, as these behaviors align with intentional family planning and reproductive decision making (20). In contexts where unplanned pregnancies account for the majority of births, as observed in this study (61.4%), the absence of reproductive agency limits women's capacity to engage proactively with health services. Partner's education emerged as a critical determinant, consistent with the evidence that husbands with higher education levels are more likely to support facility-based care and joint health decisions (21, 22). In patriarchal settings, such as Mogadishu, where household decision-making power is often concentrated in male partners, spousal education translates into health literacy, financial resource allocation toward maternal care, and reduced cultural or logistic barriers to service utilization (22). In contrast, widowed mothers face compounded vulnerabilities, including loss of partner support, constrained household income, and diminished decision-making autonomy, all of which impede their ability to navigate and complete the continuum of care. The concentration of the study population in urban and IDP settings highlights that service availability does not automatically translate into equitable access. Although health facilities may be physically proximate in Mogadishu, systemic fragmentation rooted in three decades of conflict, weak referral systems, inconsistent service quality, and persistent financial barriers creates significant dropout between successive stages of care (9, 23). Urban inequities are further amplified among IDP residents who experience displacement-related trauma, poverty, stigma, and exclusion from social support networks, all of which diminish their engagement with formal health services (9).

### Recommendation

Given the critically low continuum completion rate of 18.2% and the identified socioeconomic, educational, and reproductive determinants, the following recommendations are proposed for policymakers, program implementers, and health system stakeholders in Somalia. The Federal Ministry of Health should develop an integrated maternal health policy explicitly addressing service dropout across the continuum, with targeted attention to internally displaced populations, low-income households, and women with low or no formal education. Effective maternal health programming in Mogadishu must prioritize continuity-of-care mechanisms that reduce dropouts between antenatal care, facility delivery, and postnatal care. This includes establishing case management systems, strengthening referral networks, and deploying community health workers to facilitate transition across service contact points. Addressing the high burden of unplanned pregnancies requires integrating family planning counseling and services into antenatal and postnatal care platforms, ensuring that women have access to contraception and reproductive decision-making support throughout the maternal health continuum. Targeted interventions should focus on vulnerable populations, including widowed women, those residing in IDP camps, and households with low spousal education, by offering financial subsidies, mobile outreach services, and male engagement strategies that promote joint healthcare decision making (24). Male partner involvement programs, such as couples' counseling during antenatal care, male-friendly clinic hours, and community education campaigns, can leverage spousal influence to enhance care-seeking behaviors and reduce gender-based barriers to service access (24, 25). Urban primary healthcare systems in Mogadishu require strengthening to address cost barriers, improve service quality, and extend services equitably to marginalized populations. These efforts are essential for advancing Somalia's progress toward Sustainable Development Goal 3.1, which aims to reduce the global maternal mortality ratio to less than 70 deaths per 100,000 live births by 2030 (26).

### Strengths and limitations

This study benefits from a community-based design that captures maternal health service utilization among women with recent births, thereby minimizing the selection bias inherent in facility-based studies and reducing recall error. The inclusion of women from both urban and IDP settings provides insights into service access across heterogeneous populations within the fragile health system of Mogadishu. However, several limitations must be acknowledged. First, the cross-sectional design limits causal inference between explanatory variables and completion of the continuum of care. Second, data were self-reported and may be subject to recall bias, particularly for antenatal and postnatal care utilization. Third, the analysis was restricted to complete cases (*n* = 407) due to multicollinearity and perfect prediction among certain variables in the regression model, which may have introduced selection bias. Fourth, although a conservative proportion of 50% was used for sample size estimation to ensure adequate statistical power, this approach may differ from context-specific estimates reported in recent national surveys. Finally, the study was conducted in Mogadishu and may not be generalizable to rural or more remote regions of Somalia. Despite these limitations, this study makes an important contribution to the maternal health evidence base in Somalia, a setting where community-based research remains critically scarce, and underscores the need for integrated equity-focused interventions that address both structural health system barriers and household-level determinants of care.

## Conclusion

In conclusion, this community-based study demonstrates that over half of the women in Mogadishu completed the full maternal health continuum, yet substantial dropouts and inequities between urban and internally displaced populations persist. Pregnancy intention, spousal marital status, and partner education emerged as critical determinants, identifying key intervention points: strengthening family planning integration, implementing male engagement strategies, and targeting vulnerable populations through subsidized, accessible care. Addressing systemic fragmentation requires concurrent investment in health system infrastructure, policy reforms to reduce financial barriers, and community interventions that enhance health literacy and reproductive agencies. This evidence contributes essential data for maternal health programming in Somalia and supports progress toward Sustainable Development Goal 3.1.

## Data Availability

The raw data supporting the conclusions of this article will be made available by the authors, without undue reservation.
